# Quality of life and contact with healthcare systems among patients with psoriasis and psoriatic arthritis: results from the NORdic PAtient survey of Psoriasis and Psoriatic arthritis (NORPAPP)

**DOI:** 10.1007/s00403-019-01906-z

**Published:** 2019-03-13

**Authors:** A. Duvetorp, M. Østergaard, L. Skov, O. Seifert, K. S. Tveit, K. Danielsen, Lars Iversen

**Affiliations:** 1 Skånes Universitetssjukhus, Malmö, Sweden; 2 Department of Clinical and Experimental Medicine, Faculty of Medicine and Health Sciences, Linköping University, Linköping, Sweden; 3 Copenhagen Center for Arthritis Research, Center for Rheumatology and Spine Diseases, Rigshospitalet, Glostrup, University of Copenhagen, Copenhagen, Denmark; 4 Department of Dermatology and Allergy, Herlev and Gentofte Hospital, University of Copenhagen, Copenhagen, Denmark; 5 Division of Dermatology, Ryhov Hospital, Jönköping, Sweden; 6 Department of Dermatovenereology, Haukeland University Hospital, Bergen, Norway; 7 UiT The Arctic University of Norway, Tromsø, Norway; 8 Department of Dermatology, University Hospital of North Norway, Tromsø, Norway; 9 Department of Dermatology, Aarhus University Hospital, Aarhus, Denmark

**Keywords:** Psoriasis, Psoriatic arthritis, Quality of life, Depression

## Abstract

Psoriasis (skin psoriasis, PsO) is a chronic inflammatory condition. In about one-third of cases, the joints are affected (psoriatic arthritis, PsA). Both conditions, especially PsA, profoundly impact patients’ health-related quality of life (HRQoL). To describe the impact of psoriasis on HRQoL and patients’ contact with the healthcare system in Sweden, Denmark, and Norway, the NORdic PAtient survey of Psoriasis and Psoriatic arthritis (NORPAPP) asked 22,050 adults randomly selected in Sweden, Denmark and Norway if they had psoriasis. 1264 individuals who reported physician-diagnosed PsO/PsA were invited to the full survey; 1221 responded (74.6% diagnosed with PsO alone; 25.4% with PsA ± PsO). Respondents with PsA most frequently consulted a rheumatologist; however, 14.3% had never seen a rheumatologist. Respondents with PsO alone most frequently consulted a general practitioner and 10.7% had never seen a dermatologist (although those with severe symptoms visited dermatologists more often). Negative impacts on HRQoL were reported by 38.1% of respondents with PsO [mostly limitations on clothing (22.6%), sleep disorders (16%), and depression/anxiety (16%)] and by 73% of respondents with PsA [mostly limitations on clothing (41.8%), sports/leisure (44.0%), or daily routine (45.1%) and sleeping disorders]. Absence from work/education was more common with PsA ± PsO (51.9%) than PsO alone (15.1%). In this survey in Sweden, Denmark, and Norway, the impact of psoriasis on the respondents’ HRQoL was profound and was greater for PsA than for PsO, as was sickness absence. Sleeping disorders and depression were common and should not be overlooked.

**Electronic supplementary material:**

The online version of this article (10.1007/s00403-019-01906-z) contains supplementary material, which is available to authorized users.

## Introduction

Psoriasis (PsO) is a chronic inflammatory skin condition that, in many cases, requires life-long monitoring and management. In about one-third of cases, manifestation of the disease is not limited to the skin, but also affects the joints, causing psoriatic arthritis (PsA) [8, 20]. Both conditions, especially PsA, have a profound impact on the patient’s health-related quality of life (HRQoL), which is often underestimated [16]. An understanding of HRQoL in PsO/PsA, and its relationship with disease severity and response to treatment, is important in establishing better approaches to the care and treatment of patients with these conditions.

The Multinational Assessment of Psoriasis and Psoriatic Arthritis (MAPP) survey confirmed the profound impact of PsO and PsA on patients’ daily lives, but did not include any Nordic countries [12]. Estimates from several studies carried out since 2000 suggest that the prevalence of PsO in Scandinavia is relatively high-ranging from 3.9 up to 11.5% [5, 9, 13, 15]. The NORdic PAtient survey of Psoriasis and Psoriatic arthritis (NORPAPP) was conducted to gain a better understanding of the challenges faced by people living with PsO and PsA in Sweden, Denmark, and Norway [4]. The specific objective of this report is to describe and compare patients’ perceptions of the impact of PsO alone and PsA with or without PsO (PsA ± PsO) on their HRQoL and physician contact in these countries. The NORPAPP survey provided us with a large database of 1264 respondents with PsO and/or PsA.

## Materials and methods

Details of the methodology have been previously described [4]. The survey was conducted in Sweden, Denmark, and Norway by the international market research firm YouGov between November and December 2015, following the International Chamber of Commerce (ICC)/European Society for Opinion and Marketing Research (ESOMAR) International Code on Market, Opinion and Social Research and Data Analytics. The survey was conducted in accordance with the ethical standards required in each participating country. Briefly, an initial survey population of 22,050 adults (aged 18–74 years), randomly selected from YouGov’s panels of potential survey participants in each country, were asked if they had any type of PsO or PsA. Active sampling was used to ensure that the initial survey population was representative of the adult population in each country in terms of age and gender. All 1264 individuals who reported physician-diagnosed PsO or PsA were invited to participate in the full survey. Participants did not need to specify what type of physician had provided their diagnosis, nor did they have to be under the current care of a physician. The survey was completed via an online link sent to participants by email and the response rate was 96.6% (1221 respondents). The survey questions covered a broad range of impacts on HRQoL, expanding on the standard dermatology QoL measurement tools to include sleep disorders, hygiene, and depression/anxiety (Online Resource 1).

The respondents’ reported five-level self-perception of severity was dichotomized to ‘non-severe’ (“not severe at all” or “not particularly severe”) and ‘severe’ (“quite severe”, “very severe”, or “extremely severe”). Body surface area (BSA) was used to define the extent of PsO skin involvement. Respondents were asked to report the number of palms covered with PsO (one palm being equivalent to 1% of BSA). These two measures were only moderately correlated (Spearman’s rank correlation = 0.42) as has been reported previously [4]. Since the NORPAPP survey was designed to investigate respondents’ perspectives, the subjective severity measure was used to define subgroups in the analyses reported below.

Responses to a multipart HRQoL impact question (Online Appendix A, Q14), which were on a five-point Likert scale, were dichotomised into ‘no strong impact’ (answers 1–3 or “do not know”) and ‘strong impact’ (4 or 5). For the analysis of the frequency of absences from work/education (Online Appendix A, Q16 Have you been absent from work or school in the past 12 months due to your psoriasis/psoriatic arthritis?) responses “yes, a couple of days in the past year” and “yes, only once in the past 12 months” were grouped into ‘yes, a couple of days in the past year or less’; and responses “yes, a couple of days per week” and “yes, a couple of days per month” were grouped into ‘yes, a couple of days per month or more’.

Data from Sweden, Denmark, and Norway were analysed together as a pooled data set. Respondent data were weighted to match the demographics (gender and age) of each country. The significance of deviations in responses between subgroups based on country, diagnoses, age, sex, perceived severity of their condition, and patient group membership was assessed using Chi-squared tests and *z* tests with Bonferroni corrections (total *α* = 0.05) for comparisons of multiple answers within each question.

## Results

### Study population

Population demographics and patterns of treatment use have been reported previously [4, 19]. In brief, 48.9% were male and 55.1% were aged 45–74 years. PsO alone was diagnosed in 74.6% of respondents; the remaining respondents reported a diagnosis of PsA alone (10.3%) or PsA with PsO (PsA + PsO, 15.1%). Overall, 21.0% reported membership of a patient organisation: 14.6% of respondents with PsO alone, 46.5% with PsA alone, and 35.3% with both conditions. In their subjective judgement, most of the respondents (72.7%) with PsO alone considered their condition to be non-severe whereas 26.9% considered their condition to be severe. The severity of PsA symptoms was perceived to be greater than the severity of PsO symptoms: 38.9% of the respondents with PsA ± PsO considered their condition to be non-severe and 58.7% considered their condition to be severe. The most frequently used treatments were emollients and topical steroids, with the current use reported by 49% and 38%, respectively, of respondents with PsO and by 48% and 35% of respondents with PsA + PsO. Systemic treatment use was reported by 14.6% of respondents with PsO-only and 58.5% of respondents with PsA (including use of biologic agents by 8.1% and 31.8%, respectively).

### Contact with healthcare systems

Most of the respondents (81.7%) had seen a healthcare professional (HCP) within the previous 3 years. A substantial minority of responders with PsO alone (22.4%) had not seen an HCP for their condition in over 3 years. Corresponding figures for respondents with PsA alone and PsA + PsO were 5.6% and 6.7%.

#### Medical specialty treating PsO or PsA

**Fig. 1:**
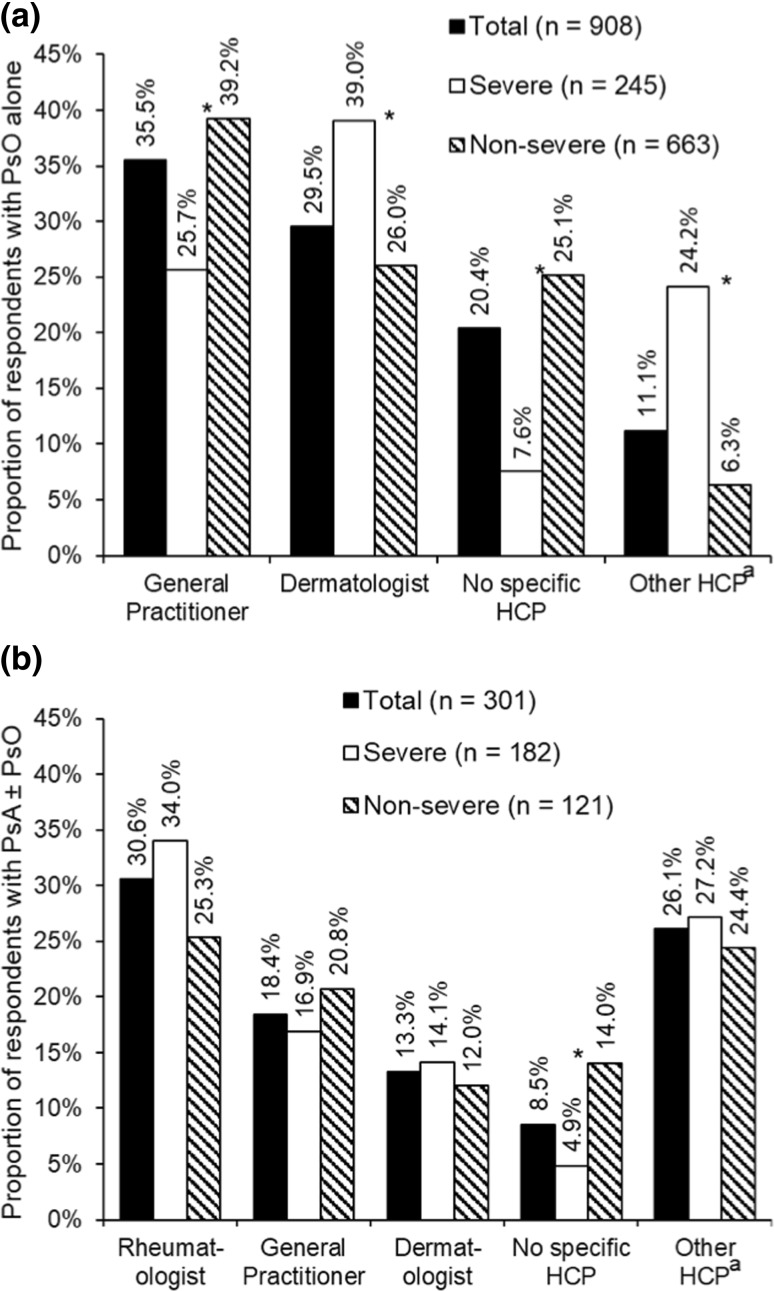
Healthcare professionals (HCPs) most frequently consulted by respondents. **a** HCP consulted for psoriasis (PsO) symptoms by respondents reporting PsO alone. **b** HCP consulted for psoriatic arthritis (PsA) symptoms by respondents reporting PsA with or without PsO (PsA ± PsO). Results are shown for all respondents in each diagnosis group and are split according to the respondent’s self-perception of the severity of their condition. (Q19. What is the medical specialty of the healthcare professional that you see most often for your…?). *Significant difference between severity groups (Bonferroni-corrected *z* tests, total *α* = 0.05). ^a^Other HCP for PsO includes: nurse, rheumatologist, allergist, unspecified HCP, physiotherapist, and orthopaedist; other HCP for PsA includes: physiotherapist, orthopaedist, nurse, and allergist. In total, 3% of respondents in each group did not know or declined to answer

Overall, respondents with PsO alone most frequently consulted a general practitioner (GP) or a dermatologist for their PsO symptoms (Fig. 1a). In Norway, it was more common to see a GP than a dermatologist, whereas, in Sweden and Denmark, it was equally common to see either a GP or a dermatologist (data not shown). Among respondents with PsO alone, those considering their PsO to be severe were more likely to see a dermatologist instead of a GP (Fig. 1a). Respondents with PsA ± PsO, irrespective of perceived severity, were most likely to report seeing a rheumatologist (Fig. 1b).

Among respondents with PsO alone, 10.7% had never seen a dermatologist. Among respondents with PsA ± PsO, 14.3% had never seen a rheumatologist. Within the previous year, 38.9% of respondents with PsO alone had seen a dermatologist and 60.9% of respondents with PsA ± PsO had seen a rheumatologist.

#### Reasons for seeing an HCP at the most recent visit

**Fig. 2:**
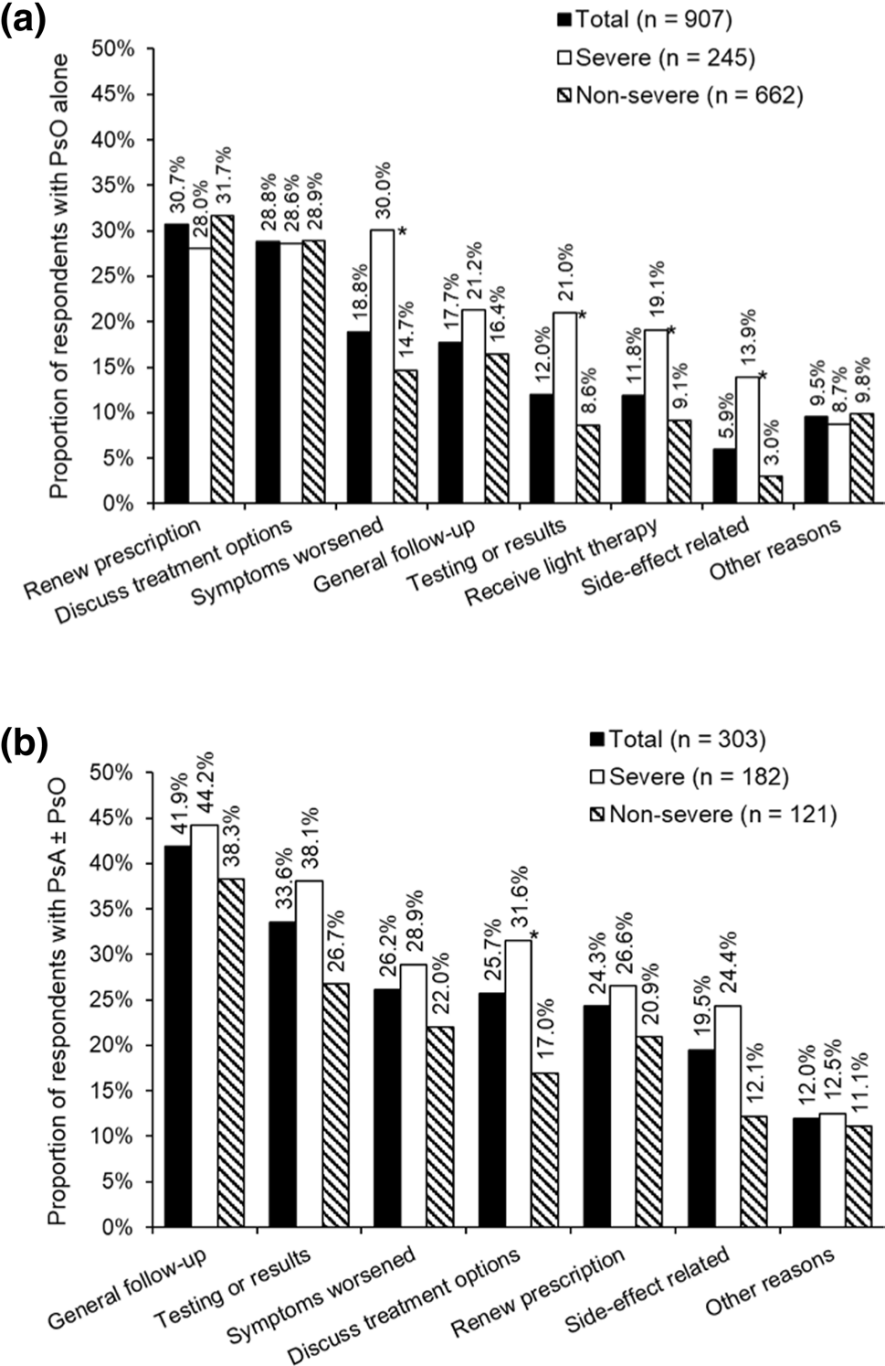
Reasons for the most recent consultation with a physician: **a** for psoriasis (PsO) symptoms, among respondents with PsO alone, **b** for psoriatic arthritis (PsA) symptoms among respondents reporting PsA with or without PsO (PsA ± PsO). Note that respondents could select more than one response to this question. (Q22/23. If you think about the last time you were in contact with a physician for your psoriasis (on skin, nails, or scalp)/psoriatic arthritis. What were the main reasons that you were in contact with the physician? *Significant difference between severity groups (Bonferroni-corrected Chi-squared tests, total *α* = 0.05). In total, 8% of respondents with PsO alone and 4% of respondents with PsA ± PsO did not know or declined to answer

For respondents with PsO alone, the most commonly reported reason for last seeing an HCP regarding PsO symptoms was renewal of a prescription, followed by the discussion of treatment options—the frequencies of these reasons were not significantly influenced by severity (Fig. 2a). Respondents with severe symptoms were more than twice as likely to have last seen an HCP for worsening of symptoms, testing or results, light therapy, and concerns regarding side-effects from medication than those with non-severe symptoms (Fig. 2a). Side-effects were mentioned by a total of 13.9% of respondents with severe PsO symptoms: 6.5% actually had side-effects and 7.4% wished to discuss possible side-effects. Side-effects were mentioned by 3.0% of respondents with non-severe symptoms; 1.0% actually had side-effects and 2.0% wished to discuss possible side-effects. Respondents who were members of a patient organisation were also significantly more likely to talk about possible side-effects than non-members (19.5% versus 3.5%, *p* < 0.05): 7.6% actually had side-effects and 11.9% wished to discuss possible side-effects.

Respondents with PsA ± PsO were most likely to have last seen an HCP regarding PsA symptoms for follow-up and testing and results (Fig. 2b). Respondents with severe PsA symptoms were significantly more likely than those with non-severe symptoms to have wanted to discuss treatment options with their physicians (Fig. 2b). Side-effects from medication were given as a reason for their most recent HCP visit by 24.4% of respondents with severe PsA symptoms: 10.0% actually had side-effects and 14.4% wanted to discuss possible side--effects. Of the respondents with non-severe symptoms, 6.3% actually had side-effects and 5.8% wished to discuss possible side-effects.

### Quality of life

#### Impact of symptoms on daily life

**Fig. 3:**
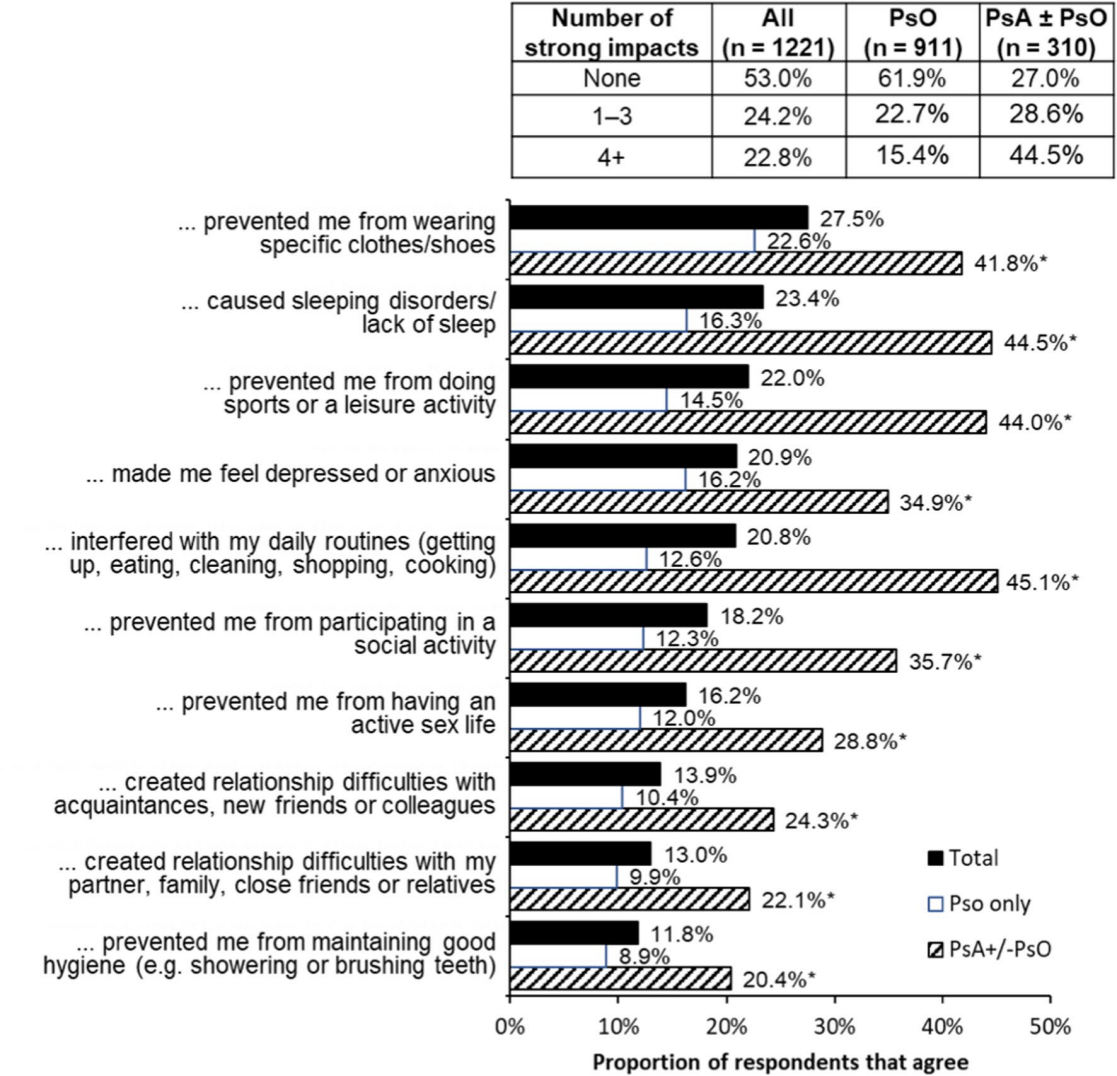
Proportion of respondents reporting that their disease had a strong negative impact on various daily activities, split by diagnosis: psoriasis (PsO) alone and psoriatic arthritis (PsA) with or without psoriasis (PsA ± PsO). The inset table shows the fraction of the respondents who reported zero, a few (1–3), or many (4 +) strong impacts. (Q14. To what extent do you agree that you have experienced the following in the past 12 months due to your psoriasis/psoriatic arthritis? The chart shows the pooled results for boxes 4 + 5 from a scale from 1 to 5 where 1 = do not agree at all and 5 = totally agree.) *Significant difference between PsO and PsA ± PsO groups (Bonferroni-corrected chi-squared tests, total *α* = 0.05). Respondents who answered “Do not know” were grouped with those not reporting a strong negative impact

Although the majority of respondents (61.9%) reporting PsO alone indicated that their disease did not strongly impact their activities of daily living, the reverse was true for respondents with PsA ± PsO; a majority (73.0%) reported at least one impact and 44.5% reported four or more impacts (Fig. 3).

For respondents with PsO alone, the most commonly reported strong negative impact was on respondent’s choice of clothing or shoes. Slightly less common, but still affecting about a sixth of the respondents with PsO alone, were sleep disorders and depression and/or anxiety (Fig. 3).

For respondents with PsA ± PsO, strong negative impacts related to daily routine, leisure/sports, sleeping disorders, and limitations on dress were reported by over 40% of respondents (Fig. 3).

Among respondents who had not seen a dermatologist or rheumatologist within the prior year, most (69.4%) reported zero impacts of their condition on daily activities. Of those respondents who had seen a dermatologist or rheumatologist within the prior year, 32.7% reported zero impacts and 38.4% reported four or more impacts on their daily activities (compared to 9.9% for those who had not seen a specialist).

#### Influence on work/career or education

**Fig. 4:**
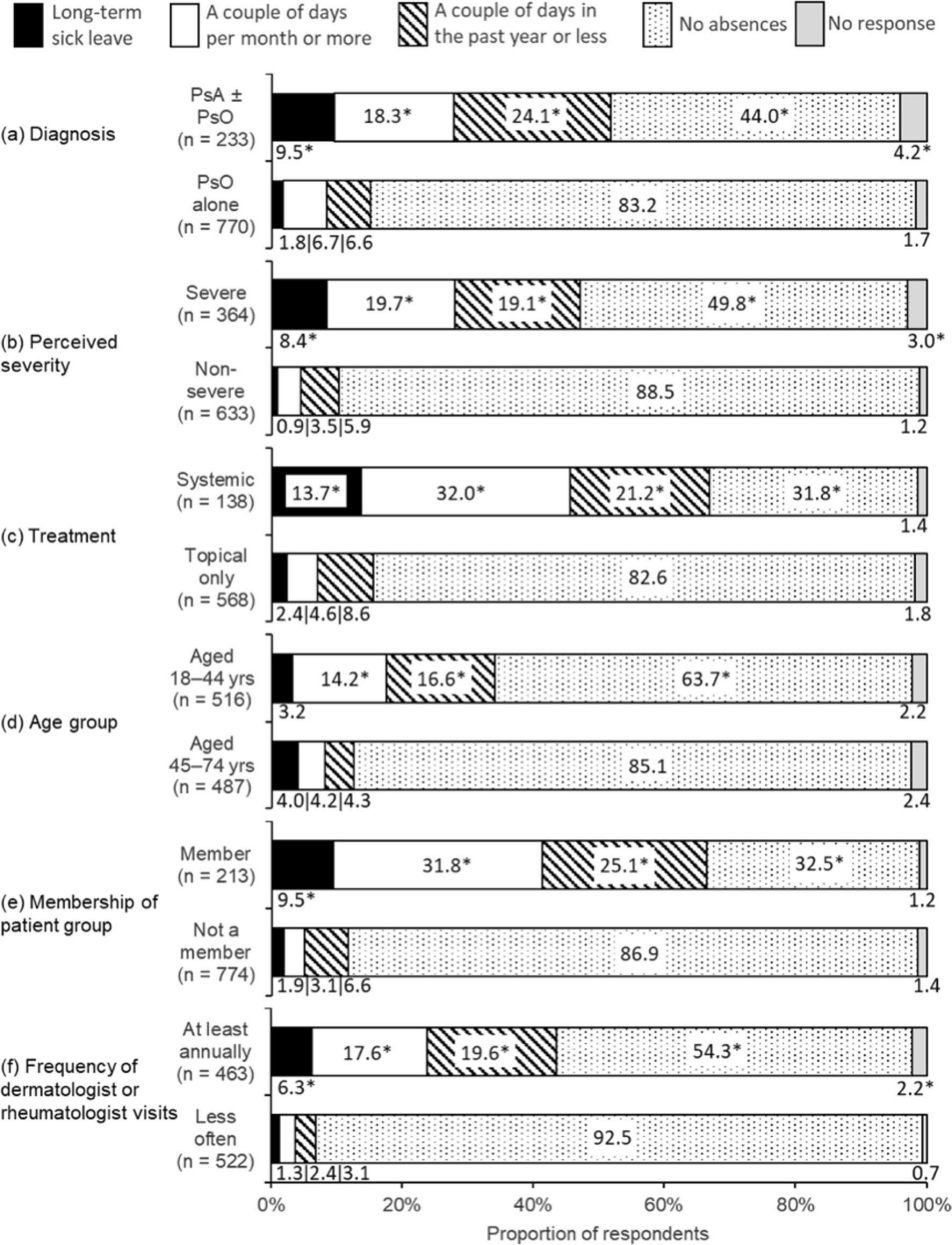
Absence from work or school (where relevant, *n* = 1003) in the previous 12 months, split by: **a** diagnosis, psoriasis (PsO) alone or psoriatic arthritis (PsA) with or without psoriasis (PsA ± PsO), **b** perceived severity, **c** treatment type, **d** age group, **e** membership of a patient group, and **f** frequency of dermatologist/rheumatologist visits. (Q16. Have you been absent from work or school in the past 12 months due to your psoriasis/psoriatic arthritis?) *Significant difference between groups in each sub-chart (Bonferroni-corrected *z* tests, total *α* = 0.05)

For those respondents for whom it was applicable (82.2% of respondents were working or studying; 94.1% of those aged 18–44, and 72.5% of those aged 45–74), the frequency of absences from work or school in the previous 12 months is shown in Fig. 4. Diagnosis significantly influenced absences from work or school (Fig. 4a). Respondents with PsA ± PsO were more likely to have reported absences than those with PsO alone. Respondents’ perception of severity was also strongly related to absences (Fig. 4b). Respondents using systemic treatments were much more likely to have been absent than those only using topical treatments (Fig. 4c). The age group of respondents also showed significant effects; those aged 18–44 years were more likely to be occasionally absent than those aged 45–74, although long-term sick-leave rates were not significantly different between the age groups (Fig. 4d; note that non-working/studying respondents were excluded from these data). Respondents who were members of patient groups were much more likely to be absent than those who were not (Fig. 4e), as were respondents who saw a dermatologist or rheumatologist at least annually (Fig. 4f). Absence rates were not significantly related gender (data not shown).

**Fig. 5:**
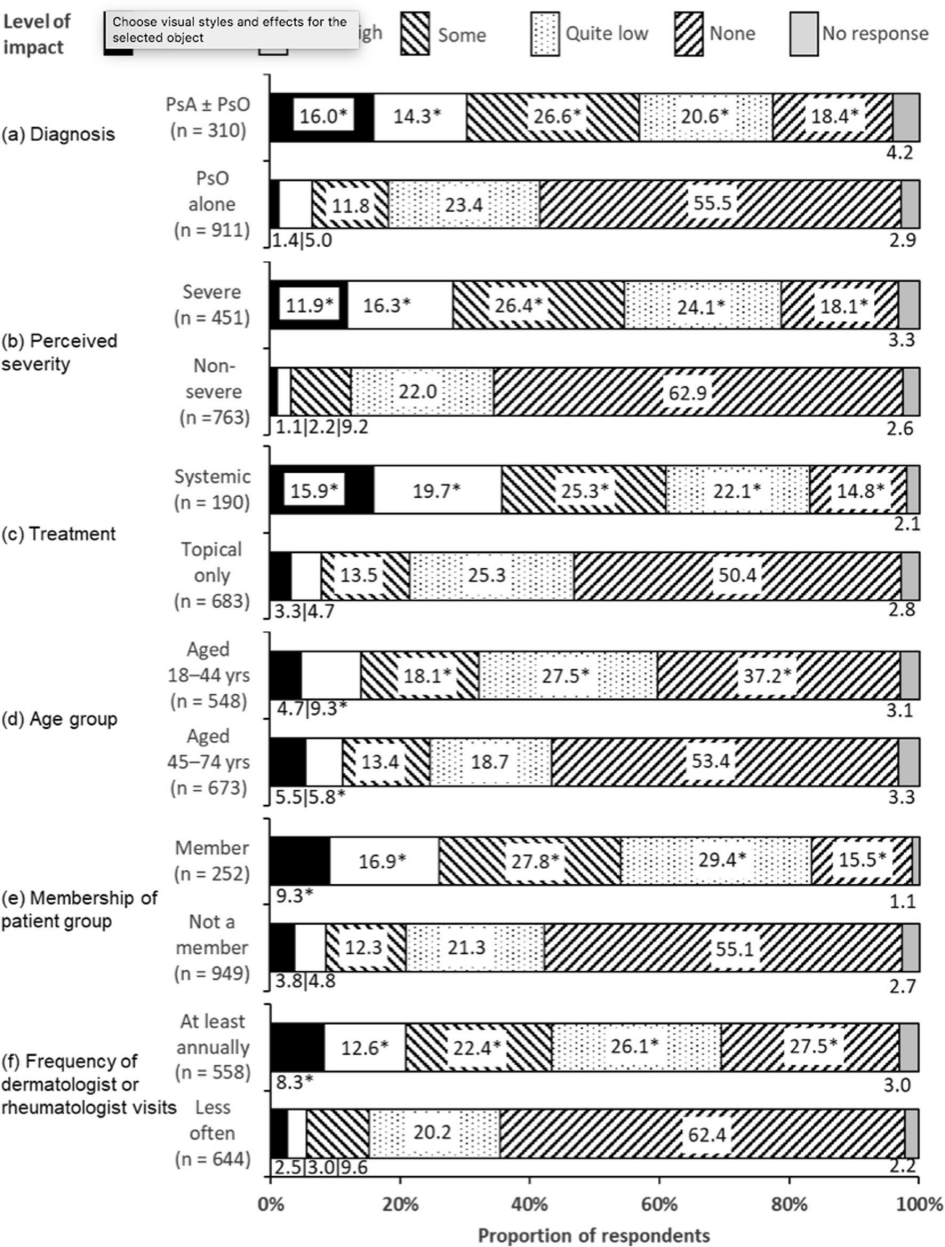
Extent to which psoriasis (PsO)/psoriatic arthritis (PsA) had negatively impacted on respondent’s work/career or education, since they first developed symptoms (n = 1221). Split by: **a** diagnosis, PsO alone or PsA with or without PsO (PsA ± PsO), **b** perceived severity, **c** treatment type, **d** age group, **e** membership of a patient group, and **f** frequency of dermatologist or rheumatologist visits. (Q17. To what extent has your psoriasis/psoriatic arthritis had a negative impact on your work/career or education, since you first developed symptoms?) *Significant difference between groups in each sub-chart (Bonferroni-corrected *z* tests, total *α* = 0.05)

Figure 5 shows the negative impact of PsO and PsA on education and work for all the respondents. Only 41.6% of respondents with PsO alone reported a negative impact, compared to 77.5% of those with PsA ± PsO (Fig. 5a). Among respondents with non-severe symptoms of PsO and/or PsA, a negative impact was reported by 34.5% (Fig. 5b). A negative impact of PsO and/or PsA was reported by a greater proportion of respondents who saw a dermatologist or rheumatologist at least annually compared with respondents who saw these HCPs less frequently (Fig. 5f).

## Discussion

Most respondents in the survey had seen a HCP within the past 3 years. The HCPs most frequently seen for PsO symptoms by respondents with PsO alone were GPs (35.5%) rather than dermatologists (29.5%). Similarly, in the MAPP study, 35% of respondents with PsO most frequently consulted a GP in both North America and in Europe [12]. A Swedish registry-based study found that nearly one-third of patients with PsO consulted only primary care physicians [14] and data from the US National PsO Foundation Survey suggests that about one quarter of patients with PsO mostly consult primary care physicians rather than a specialist [2]. Rheumatologists were most frequently consulted for PsA symptoms by approximately one-third of respondents with PsA ± PsO and this is also consistent with the MAPP study [12]. Among respondents with PsA ± PsO, the perceived severity of PsA symptoms did not significantly alter the choice of physician; respondents with or without severe symptoms reported seeing rheumatologists more frequently than GPs. We were surprised to find that 10.7% of those reporting PsO alone and 14.3% of those reporting PsA ± PsO had never seen a dermatologist or a rheumatologist, respectively.

It was noted that there was a relatively high proportion of respondents with PsA ± PsO visiting an HCP to discuss side-effects. This may be because respondents with PsA ± PsO generally had more severe disease [4], implying more potent treatment regimens with more potential side-effects; although, as we have previously reported, over one-third of respondents with severe symptoms had never discussed a systemic treatment with their physician [19]. It might also be an effect of the relatively high proportion of members of patient groups, who are likely to be more informed about potential side-effects, among respondents with PsA ± PsO.

As expected, and consistent with the previous studies in other geographical regions [1, 6, 12, 16], the reported impacts of disease on daily activities and on work or education were greater for those with PsA ± PsO compared with PsO alone, and for those with severe symptoms compared with mild symptoms. Nevertheless, more than one-third of respondents with non-severe PsO and/or PsA reported that the condition had at least some negative impact on their work or education, emphasising the importance of appropriate support even for patients with more mild conditions.

A large proportion of respondents with PsA ± PsO indicated that they suffered from sleeping disorders (44.5%) and depression and anxiety (34.9%): two impacts that are not included in the dermatology life quality index (DLQI). Thus, although it could be argued that comparison of our results with the other studies might be hampered, because we did not use a standard tool like the DLQI, the advantage of our approach is that we were able to identify two important impacts that may otherwise have been overlooked. A recent case-controlled study carried out in Norway found that almost 50% of patients with PsO suffer from substantial fatigue, which is not unusual in chronic inflammatory diseases [18]. In the MAPP study, 7% of respondents with PsA ± PsO mentioned lack of sleep as the reason for considering their condition to be severe [10]. A 2017 systematic review of the literature found that the most prevalent mental disorder in individuals with psoriasis was sleep disorders (average prevalence 62%), with anxiety (average prevalence 30.4%) and depression (average prevalence 27.6%) also frequently reported [7].

More than half of respondents (51.9%) with PsA ± PsO reported some absence from work or school over the past 12 months. This is higher than the 31.5% reported for respondents with PsA in the MAPP survey [10]. This could be a reflection of the relatively generous level of sick-leave benefits and disability pensions provided in Scandinavian countries, particularly in Sweden and Norway [3]. Indeed, overall sickness absence from employment is relatively high in Sweden and Norway; 5.2% and 5.0%, respectively for 1995–2003, compared with the European average of 2.8% [3]. Members of patient groups reported a greater impact on work/education since diagnosis (Fig. 5a) and more absences from work/education (Fig. 4a) than non-members, as did respondents who saw a HCP at least annually compared with those who visited their HCP less frequently. There may be some interaction between HCP contact, patient group membership, diagnosis, and disease severity: respondents with PsA ± PsO had more frequent HCP contact than those with PsO alone and patient group members were more likely to have a diagnosis of PsA ± PsO and were also more likely to have severe symptoms of PsO or PsA [4]. Respondents aged 45–74 years reported lower negative impacts on their careers and fewer absences from work; this is compatible with the conclusions of a recent systematic review of studies from EU countries [16].

The generalisability of the NORAPP findings is limited by factors inherent to this type of respondent survey, including a reliance on an accurate recall of facts and interpretation of questions. It is notable that a proportion of respondents had never seen a dermatologist or rheumatologist, raising the possibility that “physician-diagnosed” PsO or PsA could be wrong in some patients. A strength of the study is that, like the MAPP study, it did not rely on patient groups or clinical centres to identify or supply participants, but rather aimed to include a broadly representative cross-section of individuals living with PsO and PsA in Sweden, Denmark, and Norway. Due to the homogeneity between the Scandinavian countries when it comes to ethnicity and access to healthcare services, pooling of data was not an issue [11, 17]. The use of active sampling and weighting of results aimed to increase the representativeness of the study population. Nevertheless, the relatively high proportion of respondents who were members of patient groups (> 20%) could suggest an element of potential bias. YouGov panels are made up of a cross-section of individuals who have specifically opted in to participate in online studies; these individuals may be more likely to be members of patient groups. Given the differences that we observed between respondents who are members of patient groups and those who are not, this factor should be considered when interpreting the survey results. The difference between these two subgroups highlights that the results of surveys based entirely on respondents sourced from patient groups should not be directly compared with broader population surveys. Furthermore, patient group membership should always be reported in surveys regarding PsO and/or PsA.

In conclusion, the NORPAPP survey shows that PsO and PsA have a profound impact on the HRQoL and career/education of individuals with these conditions in Sweden, Denmark, and Norway. The impact is greater among individuals with PsA ± PsO, suggesting the importance of appropriate monitoring of patients to ensure timely diagnosis and treatment of PsA. Sleeping disorders and depression were found to affect the HRQoL of many respondents, particularly those with PsA ± PsO, and we recommend addressing these issues during consultations as they should not be overlooked. To this end, better training and education may be needed to help dermatologists, rheumatologists, and other HCPs who diagnose and manage these diseases to understand the psychological impacts of PsO and PsA.

## Electronic supplementary material

Below is the link to the electronic supplementary material.

Supplementary material 1 (PDF 204 KB)
