# Supplementary material for: Quality of life and contact with healthcare systems among patients with psoriasis and psoriatic arthritis: results from the NORdic PAtient survey of Psoriasis and Psoriatic arthritis (NORPAPP)
Source: Arch Dermatol Res. 2019 Mar 13;311(5):351–60. doi: 10.1007/s00403-019-01906-z (PMC6546664; doi:10.1007/s00403-019-01906-z)
Supplement: Supplementary file 1 — Supplementary material 1 (PDF 204 KB) [file 403_2019_1906_MOESM1_ESM.pdf]

ELECTRONIC SUPPLEMENTARY MATERIAL

Quality of life and contact with healthcare systems among patients with psoriasis and psoriatic arthritis: results from the NORdic PAtient survey of Psoriasis and Psoriatic arthritis (NORPAPP)

Journal: *Archives of Dermatological Research*

Authors: A Duvetorp[1,2], M Østergaard[3], L Skov[4], O Seifert[2,5], KS Tveit[6], K Danielsen[7,8] L Iversen[9]

Corresponding author: Lars Iversen, Department of Dermatology, Aarhus University Hospital, Aarhus, Denmark; lars.iversen@clin.au.dk

Online Resource 1: Full questionnaire

Note that only those questions relevant to each individual were presented to them.

Question 1: Do you have any type of psoriasis or psoriatic arthritis?

i) Yes, and I'm diagnosed by a physician

ii) Yes, but I'm not diagnosed by a physician

iii) No, I do not have any type of psoriasis

iv) Do not know/do not wish to answer

Question 2: Which type of psoriasis have you been diagnosed with by a physician?

i) Psoriasis on the skin, on nails or the scalp

ii) Psoriatic arthritis

iii) Both psoriasis (on skin, nails or scalp) and psoriatic arthritis

iv) Do not know/do not wish to answer

Question 3: How long after your first symptoms were you diagnosed by a physician?

Separate answers for "Psoriasis (on skin, nails or scalp)" and "Psoriatic arthritis"

i) Within one year

ii) One year after

iii) 2–4 years after

iv) 5–9 years after

v) 10–14 years after

vi) 15–19 years after

vii) 20–29 years after

viii) 30 years after or more

ix) Do not know/do not wish to answer

Question 4: If you think about the past <u>12 months</u>, how would you rate the severity of your … ?

Separate answers for "Psoriasis (on skin, nails or scalp)" and "Psoriatic arthritis"

i) Not severe at all

ii) Not particularly severe

iii) Quite severe

iv) Very severe

v) Extremely severe

vi) Do not know/do not wish to answer

Question 5: Please describe in which way your psoriasis (on skin, nails or scalp) is quite, very or extremely severe

- *Free text answer*

Question 6: Please describe in which way your psoriatic arthritis is quite, very or extremely severe

- *Free text answer*

Question 7: Which symptoms or troubles due to your psoriasis (on skin, nails or scalp) have you experienced in the past 12 months?

i) Bleeding

ii) Burning

iii) Depression or anxiety

iv) Fatigue

v) Flaking/scales

vi) Itching

vii) Pain

viii) Plaque

ix) Pustules (pus-filled blisters)

x) Redness

xi) Swollen fingers or toes (e.g. sausage digits, so called dactylitis)

xii) Tender or swollen tendons (e.g. on the heal, so called enthesitis)

xiii) Nail psoriasis

xiv) Other symptoms

xv) I have not experienced any symptoms in the past 12 months

xvi) Do not know/do not wish to answer

Question 8: Where on the body have you had symptoms of psoriasis (on skin, nails or scalp) in the past 12 months?

i) Ankles

ii) Arm pit

iii) Back/spine

iv) Chest

v) Elbow

vi) Fingers

vii) Genitals

viii) Heels

ix) Hips

x) In skin folds (inverse psoriasis)

xi) Knees

xii) Nails

xiii) Neck

xiv) Scalp

xv) Shoulders

xvi) The bend of the arm

xvii) Palm of hands or sole of feet

xviii) Toes

xix) Ears

xx) Wrist

xxi) Other body location

xxii) I have not experienced any symptoms in the past 12 months

xxiii) Do not know/do not wish to answer

Question 9: Based on the amount of psoriasis that could be covered by the palm of your hand, about how many palms would you say that you currently have across your entire body?

i) None

ii) Less than 1 palm

iii) 1–3 palms

iv) 4–9 palms

v) 10–19 palms

vi) 20 palms or more

vii) Do not know/do not wish to answer

Question 10: How often have you had relapses or flare-ups of your psoriasis (on the skin, nails or scalp) in the past three years? With relapse or flare-up, we mean periods when symptoms have worsened.

i) I've had constant symptoms, with no remission

ii) Every week

iii) Every month

iv) Every quarter

v) Every six months

vi) Every year

vii) Every two years

viii) Every three years (once in the past three years)

ix) I haven't had any flare-up or relapses in the past three years

x) Do not know/do not wish to answer

Question 11: Have you experienced pain or soreness in any of your joints?

i) Yes

ii) Yes, I have it currently

iii) Yes, in the past 12 months

iv) Yes, more than one year ago

v) No, never

vi) Do not know/do not wish to answer

Question 12: In which location is/were your joint pain or soreness most bothersome?

i) Ankles

ii) Back/spine

iii) Elbow

iv) Fingers/hands

v) Heel

vi) Sole of foot

vii) Hips

viii) Knees

ix) Neck

x) Shoulders

xi) Toes

xii) Wrist

xiii) Other body location

xiv) Do not know/do not wish to answer

Question 13: To what extent do you agree that you have experienced the following in the past 12 months due to your psoriasis?

Each statement scored from 1 (do not agree at all) to 5 (totally agree)

i) I’ve felt embarrassed or self-conscious because of my skin symptoms

ii) The topical treatment (such as ointments or creams) of my skin has been taking up too much time

i) The topical treatment (such as ointments or creams) of my skin has been inconvenient or messy

Question 14: To which extent do you agree that you have experienced the following in the past 12 months due to your psoriasis/psoriatic arthritis?

Each statement scored from 1 (do not agree at all) to 5 (totally agree)

My disease has …

i) ... caused sleeping disorders/lack of sleep

ii) .. prevented me from maintaining good hygiene (e.g. showering or brushing the teeth)

iii) ... interfered with my daily routines such as getting out of bed, eating, cleaning, shopping or cooking

iv) ... prevented me from wearing specific clothes/shoes

v) ... prevented me from participating in a social activity

vi) ... prevented me from doing sports or a leisure activity

vii) ... made me feel depressed or anxious

viii) ... prevented me from having an active sex life

ix) ... created difficulties in the relationship with my partner, family, close friends or relative

x) ... created difficulties in the relationship with acquaintances such as new friends or colleagues

Question 15: Have you experienced any trouble doing different activities in the past 12 months due to your psoriatic arthritis?

i) Yes, getting dressed, including tying shoelaces and doing buttons

ii) Yes, getting in and out of bed

iii) Yes, lifting a full cup or glass to my mouth

iv) Yes, walking outdoors on flat ground

v) Yes, washing and drying my body

vi) Yes, bending down to pick up something up from the floor

vii) Yes, turning faucets on and off

viii) Yes, getting in and out of a car

ix) Yes, having sex

x) Yes, other difficulties, what? - *Free text answer*

xi) No, I haven't had any trouble

xii) Do not know/do not wish to answer

Question 16: Have you been absent from work or school in the past 12 months due to your psoriasis/psoriatic arthritis?

i) Yes, I've been on a long-term sick leave

ii) Yes, a couple of days per week

iii) Yes, a couple of days per month

iv) Yes, a couple of days in the past year

v) Yes, only once in the past 12 months

vi) No

vii) I did not work or go to school in the past 12 months

viii) Do not know/do not wish to answer

Question 17: To what extent has your psoriasis/psoriatic arthritis had a negative impact on your work/career or education since you first developed symptoms?

i) No impact at all

ii) Quite low impact

iii) Impact

Question 18: Which types of healthcare professionals have you seen in the past three years for your … ?

Separate answers for "Psoriasis (on skin, nails or scalp)" and "Psoriatic arthritis"

i) Allergist

ii) General practitioner

iii) Dermatologist

iv) Rheumatologist

v) Nurse

vi) Physiotherapist

vii) Orthopaedist

viii) Other healthcare professional

ix) I haven’t seen any healthcare professionals

x) Do not know/do not wish to answer

Question 19: What is the medical specialty of the healthcare professional that you see most often for your …

?

Separate answers for “Psoriasis (on skin, nails or scalp)” and “Psoriatic arthritis”

i) Allergist

ii) General practitioner

iii) Dermatologist

iv) Rheumatologist

v) Nurse

vi) Physiotherapist

vii) Orthopaedist

viii) Other healthcare professional

ix) I Do not have a specific healthcare professional that I see most often

x) Do not know/do not wish to answer

i) Do not know/do not wish to answer

Question 20: When did you last see a dermatologist for your psoriasis (on skin, nails or scalp)?

i) In the past week

ii) In the past month

iii) In the past quarter

iv) In the past six months

v) In the past year

vi) In the past two years

vii) Three years ago or more

viii) I have never seen a dermatologist

ix) Do not know/do not wish to answer

Question 21: When did you last see a rheumatologist for your psoriatic arthritis?

i) In the past week

ii) In the past month

iii) In the past quarter

iv) In the past six months

v) In the past year

vi) In the past two years

vii) Three years ago or more

viii) I have never seen a rheumatologist

ix) Do not know/do not wish to answer

Question 22: If you think about the last time you were in contact with a physician for your psoriasis (on skin, nails or scalp). What were the main reasons that you were in contact with the physician? You may choose several options below.

i) Renewal of a prescription

ii) My symptoms had worsened

iii) General follow-up

iv) To talk about possible side-effects from medication

v) I had side-effects from a medication

vi) To discuss treatment options

vii) To discuss test results

viii) To take tests (such as a blood test)

ix) To receive light therapy

x) Psychological reasons (e.g. depression or anxiety)

xi) Other reason, what? - *Free text answer*

xii) Do not know/do not wish to answer

Question 23: If you think about the last time you were in contact with a physician for your psoriatic arthritis. What were the main reasons that you were in contact with the physician? You may choose several options below.

i) Renewal of a prescription

ii) My symptoms had worsened

iii) General follow-up

iv) To talk about possible side-effects from medication

v) I had side-effects from a medication

vi) To discuss treatment options

vii) To discuss test results

viii) To take tests (such as a blood test)

ix) Psychological reasons (e.g. depression or anxiety)

x) Other reason, what? - *Free text answer*

xi) Do not know/do not wish to answer

Question 24: Overall, how satisfied or dissatisfied are you with the healthcare system and treatment of your … ?

Separate answers for "Psoriasis (on skin, nails or scalp)" and "Psoriatic arthritis"

i) Very dissatisfied

ii) Neither dissatisfied nor satisfied

iii) Quite satisfied

iv) Very satisfied

v) Do not know/do not wish to answer

Question 25a: Why are you dissatisfied with the healthcare system or treatment of your psoriasis (on skin, nails or scalp)?

- *Free text answer*

Question 25b: Why are you satisfied with the healthcare's system or treatment of your psoriasis (on skin, nails or scalp)?

- *Free text answer*

Question 26a: Why are you dissatisfied with the healthcare's system or treatment of your psoriatic arthritis?

- *Free text answer*

Question 26b: Why are you satisfied with the health care's system or treatment of your psoriatic arthritis?

- *Free text answer*

Question 27: Have you ever changed physician because you were dissatisfied with the management or treatment of your …?

Separate answers for "Psoriasis (on skin, nails or scalp)" and "Psoriatic arthritis"

i) Yes, once

ii) Yes, several times

iii) No, I never had the reason to change

iv) No, I never had the option to change

v) Do not know/do not wish to answer

Question 28: Have you had a dialogue with your physician about using any of the below treatment options for your psoriasis/psoriatic arthritis?

Treatment options:

- Oral (tablet) or injectable methotrexate
- Oral (tablet) medications (other than methotrexate)
- Injectable or intravenous biologic medication

i) We discussed it but I never used it

ii) We discussed it and I used it/will use it

iii) We never discussed it

iv) Do not know/do not wish to answer

Question 29: Why did you decide not to use oral (tablet) or injectable methotrexate?

- *Free text answer*

Question 30: Why did you decide not to use an oral (tablet) medication (other than methotrexate)?

- *Free text answer*

Question 31: Why did you decide not to use an injectable or intravenous biologic medication?

- *Free text answer*

Question 32: Are you using any of the following treatments for your psoriasis/psoriatic arthritis?

Treatment options:

- Emollients (such as creams or ointments without cortisone)
- Topical cortisone treatments (topical steroids)
- Alternative treatments (e.g. from health food stores)
- Light treatment (e.g. UV treatment)
- Oral (tablet) or injectable methotrexate
- Oral (tablet) medications (other than methotrexate)
- Injectable or intravenous biologic medication
- Other treatment

i) Yes, I'm currently using it

ii) No, I used it but stopped

iii) No, I never used it

iv) Do not know/do not wish to answer

Question 33: How long did you take the oral (tablet) or injectable methotrexate for before you stopped?

i) A week or less

ii) Two to three weeks

iii) A month

iv) A quarter (three months)

v) Half a year

vi) One year

vii) Two years

viii) More than two years

ix) Do not know/do not wish to answer

Question 34: In general, how satisfied or dissatisfied are/were you with using oral (tablet) or injectable methotrexate?

i) Very dissatisfied

ii) Quite dissatisfied

iii) Neither dissatisfied nor satisfied

iv) Quite satisfied

v) Very satisfied

vi) Do not know/do not wish to answer

Question 35: Why are/were you dissatisfied with oral (tablet) or injectable methotrexate?

i) I experienced side-effects, e.g. nausea, vomiting, diarrhoea or fever

ii) I currently have/had problems with my liver

iii) I have/had to change my lifestyle too much, e.g. limit the alcohol intake or follow a specific diet

iv) I Do not/did not get enough effect

v) I Do not/did not get any effect at all

vi) I'm not/I wasn't able to keep up with my daily activities (e.g. missed work; missed family/personal time)

vii) I am/was concerned about the long-term safety

viii) It's too difficult to remember to take the medication

ix) Other

x) Do not know/do not wish to answer

Question 36: For how long did you take the injectable or intravenous biologic medication before you stopped?

i) A week or less

ii) Two to three weeks

iii) A month

iv) A quarter (three months)

v) Half a year

vi) One year

vii) Two years

viii) More than two years

ix) Do not know/do not wish to answer

Question 37: When you first were prescribed the biologic medication, was it mainly yourself requesting the treatment or was it the doctor's suggestion?

i) The doctor first suggested that I use the treatment

ii) I was the one who first suggested that I use the treatment

iii) Both the doctor and I suggested that I use the treatment

iv) None of the above

v) Do not know/do not wish to answer

Question 38: How satisfied or dissatisfied are/were you with using the biologic medication?

i) Very dissatisfied

ii) Quite dissatisfied

iii) Neither dissatisfied nor satisfied

iv) Quite satisfied

v) Very satisfied

vi) Do not know/do not wish to answer

Question 39: Why are/were you dissatisfied with the biologic medication?

i) I experienced side-effects, e.g. nausea, vomiting, diarrhoea or fever

ii) I have/ had to change my lifestyle too much, e.g. limit the alcohol intake or follow a specific diet

iii) I Do not/did not get enough effect

iv) I Do not/did not get any effect at all

v) I'm not/I wasn't able to keep up with my daily activities (e.g. missed work; missed family/personal time)

vi) I Do not like to self-inject

vii) I am/was concerned about the long-term safety

viii) It's too difficult to remember to take the medication

ix) I get/got injection-site reactions

x) Other

xi) Do not know/do not wish to answer

Question 40: How would you evaluate the following treatment types in regards to health risks of long-term use?

Treatment types

- Topical steroids, e.g. cortisone salves or creams
- Oral (tablet) or injectable methotrexate
- Oral (tablet) medications (other than methotrexate)
- Injectable or intravenous biologic medication

i) No health risks at all

ii) Quite low health risks

iii) Some health risks

iv) Quite large health risks

v) Very large health risks

vi) Do not know

Question 41: Do you think there is a good or bad range of treatment options available today for your … ?

Separate answers for "Psoriasis (on skin, nails or scalp)" and "Psoriatic arthritis"

i) Very bad range

ii) Quite bad range

iii) Neither good nor bad range

iv) Quite good range

v) Very good range

vi) Do not know

Question 42: If you think about your psoriasis/psoriatic arthritis as a whole, what is most important to you in terms of support or treatment?

- *Free text answer*

Question 43: Where do you get most of your information about psoriasis/psoriatic arthritis? You may choose a maximum of three options below.

i) Doctors

ii) Nurses

iii) Other health professionals

iv) Other patients

v) Family or friends

vi) Patient organisations

vii) Books

viii) Library

ix) Internet – search engines (such as Google)

x) Internet – specific disease-related sites

xi) Internet – forums

xii) Internet – other

xiii) Television

xiv) Radio

xv) Newspapers

xvi) Magazines

xvii) Other, what? - *Free text answer*

xviii) Do not know

Question 44: Are you a member of any patient organisation for psoriasis or psoriatic arthritis?

Sweden

i) Yes, Psoriasisförbundet

ii) Yes, Reumatikerförbundet

iii) Yes, another organisation

iv) No

v) Do not know/do not wish to answer

Norway

i) Yes, Psoriasis- og eksemforbundet

ii) Yes, Norsk Revmatikerforbund

iii) Yes, another organisation

iv) No

v) Do not know/do not wish to answer

Denmark

i) Yes, Danmarks Psoriasis Forening

ii) Yes, Gigtforeningen

iii) Yes, another organisation

iv) No

v) Do not know/do not wish to answer
